# Cryptococcosis in HIV-negative Patients with Renal Dialysis: A Retrospective Analysis of Pooled Cases

**DOI:** 10.1007/s11046-017-0163-3

**Published:** 2017-06-30

**Authors:** Nan Hong, Min Chen, Wenjie Fang, Abdullah M. S. Al-Hatmi, Teun Boekhout, Jianping Xu, Lei Zhang, Jia Liu, Weihua Pan, Wanqing Liao

**Affiliations:** 1 Shanghai Key Laboratory of Molecular Medical Mycology, Department of Dermatology, Changzheng Hospital, Second Military Medical University, Shanghai, China; 2 Department of Biology, McMaster University, Hamilton, Canada; 3 Westerdijk Fungal Biodiversity Institute, Utrecht, The Netherlands; 4 Institute of Biodiversity and Ecosystem Dynamics (IBED), University of Amsterdam, Amsterdam, The Netherlands; 5 Directorate General of Health Services, Ministry of Health, Ibri Hospital, Ibri, Oman

**Keywords:** Cryptococcosis, Renal dialysis, Pooled analysis

## Abstract

Cryptococcosis is a lethal fungal infection mainly caused by *Cryptococcus neoformans/C. gattii* species. Currently, our understanding of cryptococcosis episodes in HIV-negative patients during renal dialysis remains scarce and fragmented. Here, we performed an analysis of pooled cases to systemically summarize the epidemiology and clinical characteristics of cryptococcosis among HIV-negative patients with renal dialysis. Using pooled data from our hospital and studies identified in four medical databases, 18 cases were identified and analyzed. The median duration time of renal dialysis for peritoneal renal dialysis and hemodialysis cases was 8 months and 36 months, respectively. Several non-*neoformans/gattii* species were identified among the renal dialysis recipients with cryptococcosis, particularly *Cryptococcus laurentii* and *Cryptococcus albidus*, which share similar clinical manifestations as those caused by *C. neoformans* and *C. gattii*. Our analyses suggest that physicians should consider the possibility of the occurrence of cryptococcosis among renal dialysis recipients even when cryptococcal antigen test result is negative. The timely removal of the catheter is crucial for peritoneal dialysis patients with cryptococcosis. In addition, there is a need for optimized antifungal treatment strategy in renal dialysis recipients with cryptococcal infections.

## Background

Dialysis is a successful therapeutic process used for removing metabolic waste from the body fluids of patients with end-stage renal disease (ESRD). There are two primary types of dialysis, namely peritoneal dialysis (PD) and hemodialysis [1]. More than one million ESRD people worldwide regularly undergo renal dialysis [1, 2]. However, microbial infection remains one of the most common complications among patients receiving renal dialysis [3]. According to a retrospective study of 327,993 renal dialysis patients conducted in the USA, chronic dialysis recipients have nearly 10 times the risk of fungal infections compared to the general population [4]. The cumulative annual incidence of infection-related hospitalization during renal dialysis was 26% for children and 31% for adults in the USA [5].

In addition to bacterial and viral infections, fungal infections are becoming increasingly frequent among renal dialysis patients, with significant morbidity and mortality rates [6]. For example, fungal infections account for an estimated 5% of all PD-related peritonitis and have a higher mortality rate than bacterial PD-related peritonitis, with a mortality of 8.6–40.6% [4]. *Candida* spp. (70%) was the overwhelming microbial pathogen that caused fungal infections in patients receiving renal dialysis, followed by *Cryptococcus* spp. (6%) and *Coccidioides* spp. (4%) [4]. These data suggest that further attention should be paid to prevent or to timely diagnose fungal infections among ESRD patients during renal dialysis therapy.

The *Cryptococcus* genus, which belongs to the Basidiomycota, is the second most common group of fungal pathogens associated with renal dialysis patients [4, 7–9]. *Cryptococcus neoformans* and *C. gattii* are the main *Cryptococcus* species causing infections in humans. In contrast, *Cryptococcus laurentii*, *Cryptococcus albidus* and *Cryptococcus arboriformis* are generally regarded as saprophytes and have been rarely reported as agents causing human infections [10]. However, there has been an incremental rise in infections due to these organisms over recent decades [11]. This increase may reflect enhanced awareness of such infections, improved laboratory detection technology and a rise in the number of at-risk patients. These infections have a similar clinical presentation as those caused by *C. neoformans* or *C. gattii*, but cryptococcal antigen (CrAg) test results are frequently negative and the antifungal susceptibility patterns of these species are characterized by higher MICs [11, 12].

Despite the remarkable achievement of renal dialysis in prolonging the life of ESRD patients, the use of dialysis is problematic in some respects, such as fungal infection. Individual case reports of cryptococcosis in renal dialysis patients have been reported worldwide since 1980, and particularly since 2010. Currently, our understanding of cryptococcosis during renal dialysis is scarce and fragmented, which makes it challenging for physicians to timely diagnose and treat it among patients undergoing renal dialysis. Hence, we sought to perform a retrospective pooled analysis of the association between cryptococcosis and dialysis patients in our hospital and systematically reviewed the published reports, with a focus on epidemiology and clinical characteristics.

## Methods

### Case Collection

This study was conducted in Shanghai Changzheng hospital, a top renal dialysis center in Shanghai, China, which receives 85,000 renal dialysis cases annually. The protocol for this study was approved by the Institutional Review Board of Shanghai Changzheng Hospital (approval number 2016SL021). The cases included in the pooled analysis consisted of original cases from our hospital and published reports in electronic databases. The original cases were patients with a discharge diagnosis of “cryptococcosis” or “renal dialysis” who were admitted to our hospital between January 2001 and December 2016 and identified in the inpatient medical record database. We further systematically reviewed cases published in four major electronic literature databases: PubMed, Embase, ISI Web of Science, and Science Direct. The searches were limited to those written in English. The main search terms used were “cryptococcosis” and “dialysis,” both as MeSH terms and free text words. A definitive diagnosis of cryptococcosis was defined as the identification of positive findings upon *Cryptococcus* culture, India ink staining, histology, or a CrAg test.

### Data Collection and Statistical Analysis

After the cases were identified, the following data were extracted for (1) demographic and epidemiological data, (2) clinical manifestations and laboratory test results, (3) mycological results, and (4) treatment strategies and outcomes. SPSS (version 21, International Business Machines Corporation, Armonk, NY, USA) and Graph Pad Prism (version 5, Graph Pad Software, Inc. La Jolla, CA, USA) were used for statistical analysis. Results are presented as the means ± standard deviations (SDs) for normal data. A *P* value of less than 0.05 was considered statistically significant.

## Results

### Case Review and Analysis

**Fig. 1:**
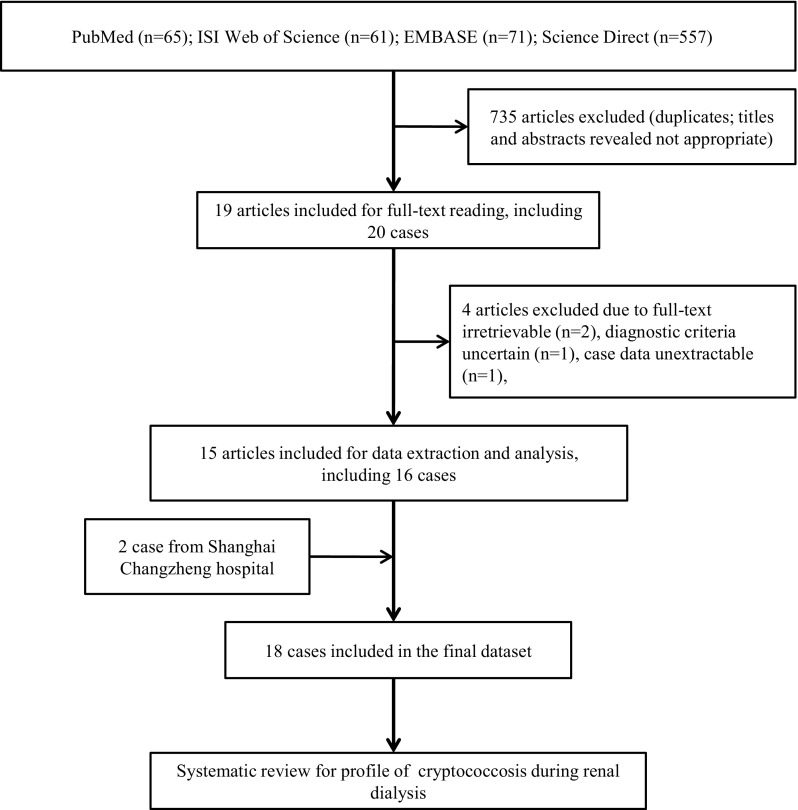
Flow diagram of the search and review processes to identify cryptococcosis cases in renal dialysis patients

A total of 754 articles were identified in the initial screen of the four international databases. Of these articles, 735 were excluded because of duplication and/or an inappropriate content. Nearly all of these pooled cases of cryptococcosis during renal dialysis were single case reports [13–27]. Among these, 16 cases reported in 15 articles with definitive diagnosis of cryptococcosis were analyzed further. In addition, we reported two renal dialysis cases with cryptococcosis who were hospitalized at our hospital. Consequently, 18 cases were pooled for analysis in the current study. The details of these cases are provided in Fig. 1.

### Demographic and Epidemiologic Characteristics

**Fig. 2:**
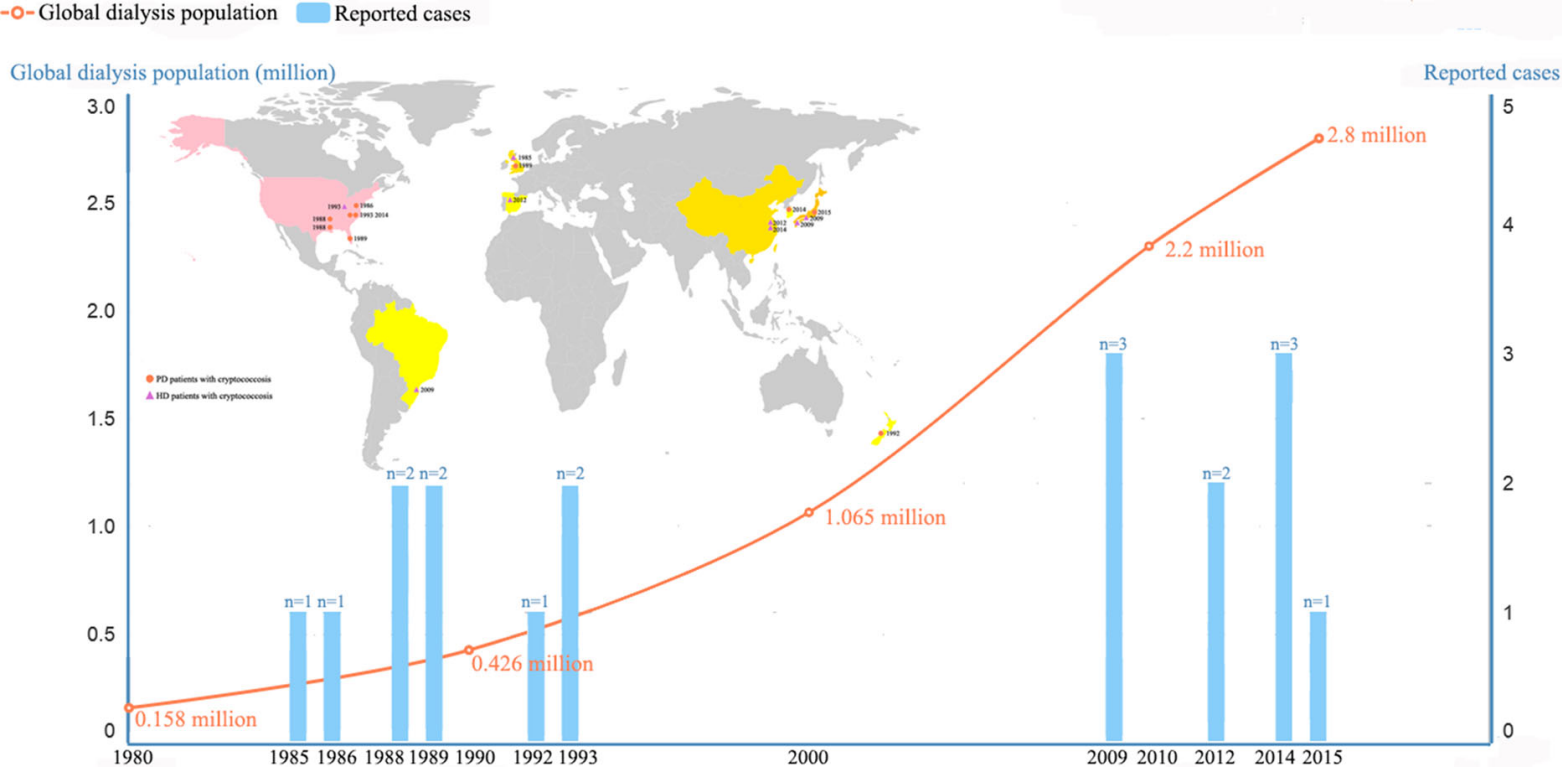
Epidemiological profiles of renal dialysis patients with cryptococcosis in the present study, 1985–2016

Among the 18 pooled cases, 10 cases were reported among PD patients and the remaining eight occurred among hemodialysis patients. The median duration of renal dialysis for PD and hemodialysis cases was 8 months (range 0.25–48 months) and 36 months (24–228 months), respectively. One PD-patient died because of cryptococcosis, and two patients with hemodialysis died because of cryptococcosis during renal dialysis therapy. All of the pooled patients were reported from medical centers in upper-middle to high-income countries (classification based on World Bank data: http://data.worldbank.org/about/country-and-lending- groups), with a male: female gender ratio of 13:5. Half of the pooled cases were reported since 2009. Most of the pooled cases were reported in the USA (38.9%, 7/18), followed by Japan (16.7%, 3/18), China (16.7%, 2/18), UK (16.7%, 2/18), Spain (5.6%, 1/18), Korea (5.6%, 1/18), Brazil (5.6%, 1/18), and New Zealand (5.6%, 1/18). The age range containing most patients was 51–60 years (27.8%, 5/18), and most patients were adults (aged ≥ 16 years old; 88.9%, 16/18). Details of the demographic and epidemiologic characteristics of the pooled cases are provided in Table 1 and Fig. 2.

**Table 1:** Epidemiological characteristics of cryptococcosis during renal dialysis, 1985–2016

Case	Date	Sex/age	Dialysis type	Geographical location	Pigeon contact	Previous dialysis duration (months)	Prognosis	References
1	1986	M/24	PD	Jacksonville, USA	ND	12	Cured	[18]
2	May 1988	F/16	PD	Little Rock, USA	ND	0.25	Cured	[20]
3	May 1988	F/49	PD	Little Rock, USA	ND	0.5	Died	[20]
4	May 1989	M/52	PD	Manchester, UK	ND	7	Cured	[19]
5	Nov 1989	F/13	PD	Tampa, USA	ND	12	Cured	[16]
6	1992	F/50	PD	Porirua, New Zealand	ND	8	Cured	[21]
7	Oct 1993	M/48	PD	New York, USA	ND	6	Cured	[22]
8	Mar 2014	M/57	PD	New York, USA	Neg	48	Cured	[14]
9	Jul 2014	M/58	PD	Seoul, Korea	ND	10	Cured	[15]
10	Apr 2015	M/32	PD	Osaka city, Japan	ND	36	Cured	[13]
11	Aug 1985	M/22	Hemodialysis	Bristol, UK	ND	24	Cured	[23]
12	Sep 1993	M/37	Hemodialysis	Philadelphia, USA	ND	228	Cured	[17]
13	Aug 2009	F/60	Hemodialysis	Curitiba, Brazil	ND	48	Died	[24]
14	Jan 2009	M/49	Hemodialysis	Matsuyama, Japan	ND	0.25	Died	[27]
15	Sep 2009	M/64	Hemodialysis	Okinawa, Japan	Pos	60	Cured	[26]
16	Jun 2012	M/53	Hemodialysis	Oviedo, Spain	ND	24	Cured	[25]
17	May 2012	M/36	Hemodialysis	Shanghai, China	Neg	24	Cured	This study
18	Aug 2014	M/45	Hemodialysis	Shanghai, China	Neg	96	Cured	This study

*PD* peritoneal dialysis, *M* male, *F* female, *ND* no data

### Clinical Manifestations

Among the 10 PD cases, most cryptococcal infections occurred in the abdomen ascites (60%, 6/10), followed by two cases involving both the abdomen ascites and cerebrospinal fluid (CSF), one case involving the abdomen ascites and blood, and one case involving the abdomen ascites, blood and CSF. Fever (70.0%, 7/10), cloudy dialysate (70.0%, 7/10), and abdominal pain (50.0%, 5/10) were the most common clinical features. Remarkably, bacterial peritonitis was diagnosed frequently (30.0%, 3/10) in our pooled PD patients. The most common bacterial pathogens in this study were *Aeromonas hydrophila*, *Staphylococcus epidermidis*, and *Streptococcus* group D.

**Fig. 3:**
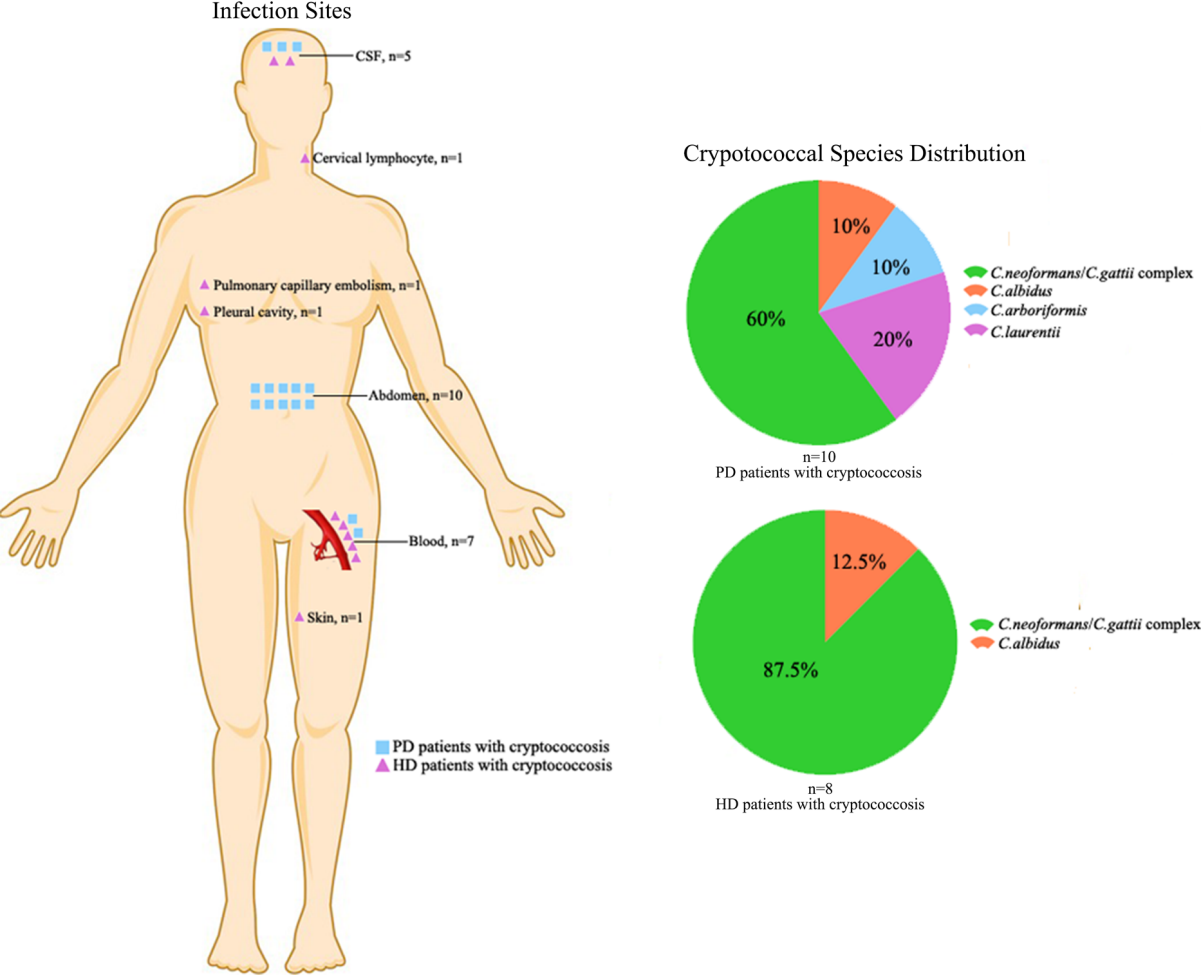
Clinical characteristics and the related cryptococcus species in the pooled renal dialysis patients

In contrast, the infection sites were variable among the 8 hemodialysis patients, including blood (*n* = 5), cervical lymphocytes (*n* = 1), pleural cavity (*n* = 2), pulmonary capillary embolism (*n* = 1), and skin (*n* = 1). Two patients were each infected at two body sites. Fever (50.0%, 4/8) was the most common clinical feature, followed by headache (25.0%, 2/8), cough (25.0%, 2/8), pleural effusion (25.0%, 2/8), cervical lymphadenopathy (12.5%, 1/8), and erythematous lesions (12.5%, 1/8). Details of the clinical manifestations are shown in Table 2 and Fig. 3.

**Table 2:** Clinical characteristics of cryptococcosis during renal dialysis

Cases	Affected site	Main manifestation	Previous antibiotics	References
1	Abdomen	Abdominal pain	Cefadyl	[18]
2	Abdomen + blood + CSF	Fever	ND	[20]
3	Abdomen + CSF	Abdominal pain + fever	ND	[20]
4	Abdomen + blood	Cloudy dialysate + fever	ND	[19]
5	Abdomen	Abdominal pain + cloudy dialysate + fever	Vancomycin	[16]
6	Abdomen	cloudy dialysate	ND	[21]
7	Abdomen + CSF	Cloudy dialysate + fever	Neg	[22]
8	Abdomen	Abdominal pain + cloudy dialysate	Vancomycin	[14]
9	Abdomen	Abdominal pain + cloudy dialysate + fever	Imipenem	[15]
10	Abdomen	Cloudy dialysate + fever	Neg	[13]
11	Cervical lymphocyte + blood	cervical lymphadenopathy + fever	ND	[23]
12	Pleural cavity	Cough + fever	ND	[17]
13	Blood	Fever	ND	[24]
14	Pulmonary capillary embolism	Severe hypoxia + dyspnea	Vancomycin	[27]
15	Pleural cavity	Cough	ND	[26]
16	Skin + blood	Cutaneous ulceration	ND	[25]
17	Blood + CSF	Headache	ND	This study
18	Blood + CSF	Headache + fever	ND	This study

*CSF* cerebrospinal fluid, *ND* no data

### Diagnostic Profile

Regarding the diagnostic profiles of the 18 pooled cases, 16 were diagnosed using cultures, 12 were diagnosed using CrAg tests, and two cases were diagnosed using India ink staining and histology. All cases (100%, 4/4) caused by non-*C. neoformans*/*C. gattii* were CrAg-negative. Notably, multiple *Cryptococcus* species were observed in the 16 cases diagnosed using cultures, including *C. neoformans* (68.7%, 11/16), *C. laurentti* (12.5%, 2/16), *C. albidus* (12.5%, 2/16), and *C. arboriformis* (6.3%, 1/16). The details are provided in Table 3.

**Table 3:** Diagnosis profile of cryptococcosis during renal dialysis

*Cryptococcus* species	Serum CrAg	Diagnostic evidence	References
*C. laurentii*	Neg	Culture	[16]
*C. albidus*	Neg	Culture	[14]
*C. arboriformis*	Neg	Culture	[15]
*C. laurentii*	Neg	Culture	[13]
*C. albidus*	ND	Culture	[17]
*Cryptococcus* spp.	ND	Culture	[18]
*C. neoformans*/C. *gattii* complex	>1:12000	Culture	[19]
*C. neoformans*/*C.gattii* complex	1:512	Culture	[20]
*C. neoformans*/*C. gattii* complex	ND	Culture	[20]
*C. neoformans* var. *neoformans*	ND	Culture	[21]
*C. neoformans*/*C. gattii* complex	1:10000	Culture	[22]
*C. neoformans*/*C. gattii* complex	Neg	Histology	[23]
*C. neoformans*/*C. gattii* complex	ND	Culture	[24]
*C. neoformans* var. *neoformans*	1:2048	Culture + histology	[25]
*C. neoformans*/*C. gattii* complex	1:320	Culture	This study
*C. neoformans*/*C. gattii* complex	1:5120	Culture	This study
*C. neoformans*/*C. gattii* complex	Neg	Culture	[26]
*Cryptococcus* spp.	ND	Microscopic examination	[27]

*Cr Ag* cryptococcal antigen, *ND* no data

### Treatment and Outcome

The antifungal therapies used to treat the pooled cases were highly variable: amphotericin B (AmB) in combination with 5-fluorocytosine (5-FC) was used in seven cases, followed by AmB alone in six cases, AmB in combination with voriconazole (VCZ) in one case, AmB in combination with fluconazole (FCZ) in one case, VCZ alone in one case, VCZ in combination with 5-FC in one case, and ketoconazole (KCZ) in combination with FCZ in one case. In addition, catheters were removed from all the PD patients (100%, 10/10) after the identification of cryptococcosis, whereas only one hemodialysis patient (12.5%, 1/8) had the catheter removed. The details are provided in Table 4.

**Table 4:** Treatment profile of cryptococcosis during renal dialysis

Case	Removal of catheter	Antifungal treatment	Outcome	References
1	Yes	AmB (cumulative dose, 0.3 g)	Switching to permanent hemodialysis	[18]
2	Yes	AmB (cumulative dose, 2 g)	Cured	[20]
3	Yes	AmB + 5-FC	Died	[20]
4	Yes	AmB (cumulative dose, 1.4 g) + 5-FC (cumulative dose, 55 g)	Switching to permanent hemodialysis	[19]
5	Yes	MCZ × 3 days + AmB (cumulative dose, 0.5 g)	Cured	[16]
6	Yes	KCZ 400 mg/day × 2 days + FCZ 400 mg/day × 5 days	Cured	[21]
7	Yes	AmB 0.5 mg/kg/day × 6 weeks +5-FC × 4 weeks	Switching to permanent hemodialysis	[22]
8	Yes	(FCZ 200 mg/day + CPF 50 mg/day) × 5 days + AmB liposomal 400 mg/day × 7 days	Switching to permanent hemodialysis	[14]
9	Yes	FCZ200 mg/day × 6 days + AmB 0.5 mg/kg/day × 4 weeks	Switching to permanent hemodialysis	[15]
10	Yes	VCZ 500 mg × 8 days	Cured	[13]
11	No	AmB (cumulative dose, 2.5 g) + 5-FC 50 mg/kg × 6 weeks	Cured	[23]
12	No	AmB (cumulative dose, 1.9 g)	Cured	[17]
13	Yes	AmB 0.5 mg/kg/day × 6 weeks	Died	[24]
14	ND	ND	Died	[27]
15	No	(AmB + 5-FC) × 9 days + FCZ × 24 weeks	Cured	[26]
16	No	VCZ 400 mg/d × 10 days + AmB 100 mg/day × 4 days +	Cured	[25]
17	No	(VCZ 0.4 g/day + 5-FC 4 g/day) × 2 weeks	Cured	This study
18	No	(AmB 30 mg/days + 5-FC 4.5 g/day) × 4 weeks	Cured	This study

*AmB* Amphotericin B, *VCZ* voriconazole, *FCZ* fluconazole, *KCZ* ketoconazole, *MCZ* miconazole, *5-FC* 5-fluorocytosine, *CPF* caspofungin, *ND* no data

## Discussion

Previous studies have shown that cryptococcosis can occur in HIV-negative patients [28, 29]. However, a detailed profile of cryptococcosis occurring after renal dialysis is difficult to determine. Recent studies suggested that non-*C*. *neoformans*/*C*. *gattii* species, which have higher MICs of current antifungal drugs, accounted for a large proportion of cryptococcal infections among renal dialysis patients [13–17]. Hence, cryptococcosis in renal dialysis patients should be further studied due to our poor understanding of this infection.

The current study showed that male adult patients were dominant among renal dialysis recipients with cryptococcosis, which is consistent with previous studies [30, 31]. A considerable number of pooled cases (5/18) occurred in the past 5 years, which is likely to be related to the increasing number of renal dialysis patients worldwide [3]. Most of the current cases were collected from medical centers in major cities worldwide, including New York in the USA, Osaka in Japan, and Shanghai in China [13, 14, 22]. Thus, the availability of modern diagnostic tools might be relevant to the relatively high level of diagnosed cryptococcal infections in these cities. Although HIV infection is the most common underlying risk factor for cryptococcal infection [11, 12], none of the renal dialysis recipients with cryptococcosis were reported to be HIV-positive in the current study. However, we cannot neglect the risk of cryptococcosis among renal dialysis recipients who also have HIV infections.

The current survey found several different characteristics between PD and hemodialysis patients with cryptococcosis. For example, the duration of exposure to renal dialysis is directly related to the risk of septicemia [32], because a longer duration of renal dialysis may have a cumulative detrimental effect on immunity. In the current study, the median duration of hemodialysis was nearly threefold longer than that of PD cases. However, there were nearly twice as many reported cases of cryptococcosis in PD patients than hemodialysis patients, which indicated that peritoneal dialysis patients might have a higher risk for cryptococcosis compared with hemodialysis patients. Remarkably, cryptococcal infections tend to be localized in the abdomen of PD patients (60.0%, 6/10). In contrast, the sites of cryptococcal infection vary among hemodialysis patients, including skin, lungs, and blood. Moreover, the current data suggest that several PD patients (40.0%, 4/10) had previously been treated for bacterial peritonitis. This finding is consistent with a study [33] encompassing 66 centers in Australia over a 4-year period, which indicated that previously treated bacterial peritonitis is a major risk factor for fungal peritonitis.

*Cryptococcus neoformans* and *C. gattii* species are responsible for almost all human cryptococcal infections [7]. Other cryptococcal species were traditionally considered to be nonpathogenic [11, 12]. However, cases of cryptococcosis caused by non-*C*. *neoformans*/*C*. *gattii* have increased significantly since the 1970s, including in renal dialysis patients. The results of the current study also revealed that non-*neoformans*/*gattii* cryptococcal species account for a considerable proportion (approximately 27.8%, 5/18) of the cryptococcal species among renal dialysis patients [13–17], particularly *C. laurentii* or *C. albidus*. This observation is consistent with a recent global review of 44 previously published cases [11]. Notably, the current results suggest that CrAg tests are ineffective for the diagnosis of cryptococcosis caused by non-*C*. *neoformans*/*C*. *gattii* species, although this test is considered as a powerful tool for the diagnosis of cryptococcal infections caused by *C*. *neoformans* or *C*. *gattii* species [34–36]. The discrepancy in the sensitivity of CrAg test might be related to the significant phylogenetic divergence between non-*C*. *neoformans*/*C*. *gattii* species and *C*. *neoformans*/*C*. *gattii* species complex [8, 9]. Indeed, recent phylogenetic studies have reclassified *C. laurentii* to *Papiliotrema laurentii*, *C. albidus* to *Naganishia albida* and *C. arboriformis* to *Cutaneotrichosporon arboriformis* [8, 9]. Another potential cause of the negative CrAg test for infections caused by *C. laurentii* and *C. albidus* might be related to a lower organism burden in cryptococcemia caused by these non-*C*. *neoformans*/*C*. *gattii* species [11]. Thus, a negative CrAg test result cannot rule out cryptococcal infections caused by non-*C*. *neoformans*/*C*. *gattii* species [37].

Regarding the treatment of cryptococcosis among dialysis patients in this study, the catheter was removed from most PD patients (91%) after the identification of a cryptococcal infection. This is consistent with a recent retrospective review indicating that catheters should be removed immediately and certainly within 24 h after the identification of fungi in PD patients with fungal peritonitis [38]. In contrast, only one hemodialysis patient had the catheter removed. Moreover, it has been shown that the risks of recurrent fungal peritonitis and death were lowest when the catheter removal is combined with antifungal therapy compared with either intervention alone [33]. In addition, multiple antifungal agents were used to treat cryptococcosis patients with renal dialysis in the current study, including AmB, FCZ, VCZ, KCZ, and flucytosine. Among these, AmB is the most frequently used antifungal drug, which is consistent with the 2010 guidelines for the treatment of cryptococcosis [39]. Treatment with AmB and FCZ is the previous recommendations for cryptococcal infections caused by non-*C*. *neoformans*/*C*. *gattii* species, including *C. albidus*, *C. laurentii*, *C. uniguttulatus* and *C. curvatus*, according to the results of an in vitro susceptibility study [12]. In addition, recent susceptibility testing of *C. arboriformis* revealed that the isolate was susceptible to AmB and FCZ, with intermediate sensitivity to flucytosine [15]. Further studies on renal dialysis recipients with cryptococcosis are needed to develop more effective and consistent treatment strategies.

In conclusion, cryptococcosis is a neglected complication among renal dialysis recipients and has shown an increasing tendency in recent years. Multiple species, such as *C. albidus*, *C. laurentii* and *C. arboriformis*, were observed with nonspecific clinical manifestations. Physicians should consider the possibility of cryptococcosis among renal dialysis recipients, even for those with a negative CrAg test result. The timely removal of the catheter was shown to be crucial for the successful recovery of PD patients with cryptococcosis. Optimizing antifungal treatment strategy in renal dialysis recipients with cryptococcal infections is needed.
